# Supplementary material for: Occupational Exposure Assessment to Antineoplastic Drugs in Nine Italian Hospital Centers over a 5-Year Survey Program
Source: Int J Environ Res Public Health. 2022 Jul 14;19(14):8601. doi: 10.3390/ijerph19148601 (PMC9321125; doi:10.3390/ijerph19148601)
Supplement: Supplementary file 1 [file ijerph-19-08601-s001.zip › ijerph-1756279-supplementary.pdf]

	PHARMACY AREAS							
Years	CP				GEM			
	Wipes	Pads	Odds Ratio (95% CI)	pvalue	Wipes	Pads	Odds Ratio (95% CI)	pvalue
2016	37 (42)	1 (5.6)	0.08 (0.01 to 0.64)	0.017	32 (36.4)	5 (27.8)	0.67 (0.22 to 2.06)	0.488
2017	23 (24)	2 (11.1)	0.4 (0.08 to 1.86)	0.24	34 (35.4)	7 (38.9)	1.16 (0.41 to 3.27)	0.778
2019	46 (43.8)	8 (38.1)	0.79 (0.3 to 2.06)	0.63	34 (32.4)	7 (33.3)	1.04 (0.39 to 2.82)	0.932
2020	79 (75.2)	2 (11.1)	0.04 (0.01 to 0.19)	<0.001	87 (82.9)	6 (33.3)	0.1 (0.03 to 0.31)	<0.001
2021	64 (67.4)	0 (0)	ND	ND	76 (80)	10 (55.6)	0.31 (0.11 to 0.9)	0.031
2016-2021	249 (50.9)	13 (14.0)	0.16 (0.08 to 0.29)	<0.001	263 (53.8)	35 (37.6)	0.52 (0.33 to 0.82)	0.005
	5-FU				Pt			
	Wipes	Pads	Odds Ratio (95% CI)	pvalue	Wipes	Pads	Odds Ratio (95% CI)	pvalue
2016	19 (21.6)	5 (27.8)	1.4 (0.44 to 4.41)	0.569	41 (46.6)	4 (22.2)	0.33 (0.1 to 1.07)	0.065
2017	19 (19.8)	6 (33.3)	2.03 (0.67 to 6.09)	0.209	37 (38.5)	1 (5.6)	0.09 (0.01 to 0.73)	0.024
2019	24 (22.9)	10 (47.6)	3.07 (1.16 to 8.09)	0.023	27 (25.7)	4 (19)	0.68 (0.21 to 2.2)	0.519
2020	57 (54.3)	2 (11.1)	0.11 (0.02 to 0.48)	0.004	45 (42.9)	1 (5.6)	0.08 (0.01 to 0.61)	0.015
2021	21 (22.1)	5 (27.8)	1.36 (0.43 to 4.24)	0.601	65 (68.4)	4 (22.2)	0.13 (0.04 to 0.43)	0.001
2016-2021	140 (28.6)	28 (30.1)	1.07 (0.66 to 1.74)	0.773	215 (44.0)	14 (15.1)	0.23 (0.12 to 0.41)	<0.001

	PATIENT CARE UNITS							
Years	CP				GEM			
	Wipes	Pads	Odds Ratio (95% CI)	pvalue	Wipes	Pads	Odds Ratio (95% CI)	pvalue
2016	158 (68.1)	12 (16)	0.09 (0.05 to 0.18)	<0.001	156 (67.2)	13 (17.3)	0.1 (0.05 to 0.2)	<0.001
2017	104 (44.1)	5 (7.2)	0.1 (0.04 to 0.26)	<0.001	163 (69.1)	17 (24.6)	0.15 (0.08 to 0.27)	<0.001
2019	159 (64.4)	2 (4.4)	0.03 (0.01 to 0.11)	<0.001	152 (61.5)	8 (17.8)	0.14 (0.06 to 0.3)	<0.001
2020	162 (76.8)	4 (12.1)	0.04 (0.01 to 0.12)	<0.001	164 (77.7)	12 (36.4)	0.16 (0.08 to 0.36)	<0.001
2021	193 (68.4)	4 (6.7)	0.03 (0.01 to 0.09)	<0.001	217 (77)	6 (10)	0.03 (0.01 to 0.08)	<0.001
2016-2021	776 (64.2)	27 (9.6)	(0.04 to 0.09)	<0.001	852 (70.5)	56 (19.9) 0.1	(0.08 to 0.14)	<0.001
	5-FU				Pt			
	Wipes	Pads	Odds Ratio (95% CI)	pvalue	Wipes	Pads	Odds Ratio (95% CI)	pvalue
2016	57 (24.6)	5 (6.7)	0.22 (0.08 to 0.57)	0,002	182 (78.4)	5 (6.7)	0.02 (0.01 to 0.05)	0,001
2017	73 (30.9)	7 (10.1)	0.25 (0.11 to 0.58)	0,001	170 (72)	6 (8.7)	0.04 (0.02 to 0.09)	0,001
2019	103 (41.7)	4 (8.9)	0.14 (0.05 to 0.39)	<0.001	124 (50.2)	0 (0)	NA	NA
2020	115 (54.5)	2 (6.1)	0.05 (0.01 to 0.23)	<0.001	130 (61.6)	1 (3)	0.02 (0 to 0.15)	0,001
2021	53 (18.8)	2 (3.3)	0.15 (0.04 to 0.63)	<0.001	218 (77.3)	2 (3.3)	0.01 (0 to 0.04)	0,001
2016-2021	401 (33.2)	20 (7.1)	0,06 (0.1 to 0.25)	<0.001	824 (68.2)	14 (5)	0.02 (0.01 to 0.04)	<0.001

Table S1. Statistical parameters and Odds Ratios values in pharmacy areas and patient care units.
